# The challenges of COVID-19 in the Brazilian Amazonian communities and the importance of seroepidemiological surveillance studies

**DOI:** 10.1186/s12939-020-01256-7

**Published:** 2020-08-15

**Authors:** Antonio Carlos Rosário Vallinoto, Maria Karoliny da Silva Torres, Mariana Cayres Vallinoto, Izaura M. V. Cayres Vallinoto

**Affiliations:** 1 Laboratório de Virologia, Universidade Federal do Pará, Belem, Para Brazil; 2 Programa de Pós-graduação em Biologia de Agentes Infecciosos e Parasitários, Universidade Federal do Pará, Belem, Para Brazil; 3 Centro Universitário do Estado do Pará, Belém, Pará Brazil

## Abstract

The emergence of the severe acute respiratory syndrome coronavirus 2 (SARS-CoV-2) has alarmed the world with its high rate of transmission and the ability to cause severe and fatal disease. The impact of this pandemic may be even greater in populations where the absence of health services is a chronic aspect, as reported with populations living in the Brazilian Amazon. In this article, we address the perspective of possible impacts of the pandemic on these populations and the importance of conducting seroepidemiological surveillance studies.

## Background

The emergence of a new Severe Acute Respiratory Syndrome Coronavirus 2 (SARS-CoV-2) in Wuhan, China, in late 2019, coupled with its rapid spread to Chinese provinces and to more than 100 countries on all continents, has led the World Health Organization (WHO) to define the situation as a new pandemic [1]. The rapid spread of SARS-CoV-2 has forced all nations in the world to mobilize a variety of efforts to restrain the spread of the virus, such as: (i) put in place strong measures to detect disease early, (ii) isolate and treat cases, (iii) trace contacts, (iv) promote social distancing measures commensurate with the risk, (v) conducting studies on the severity and transmissibility of the virus, (vi) increase in the hospital network, especially in the number of intensive care beds exclusively for patients with COVID-19, and (vii) sharing data and biological material [1]; however, given the many uncertainties regarding the transmissibility, pathogenicity and virulence of this new infectious agent, the effectiveness of these efforts is still unknown. SARS-CoV-2 is the seventh coronavirus known to infect human beings (HCoVs - Human Coronavirus); SARS-CoV (Severe Acute Respiratory Syndrome Coronavirus), MERS-CoV (Middle East Respiratory Syndrome Coronavirus) and SARS-CoV-2 can cause serious diseases, while HCoV-HKU1, HCoV-NL63, HCoV-OC43 and HCoV-229E are strains associated with mild symptoms [2].

Worldwide, the diagnosis of SARS-CoV-2 infection is mainly based on moderate and severe symptomatic cases requiring hospitalization. Asymptomatic cases, which are undocumented, represents a critical epidemiological feature in the modulation of the potential spread of an emerging respiratory virus such as SARS-CoV-2 [3]. These infections go unnoticed and, depending on their capacity for transmissibility and contagion, can expose a much larger portion of the population to the virus than would normally occur [3]. The current situation of the pandemic exceeds five million cases and 340 thousand deaths, with a mortality rate of approximately 5%; however, these figures are underestimates [4], mainly due to asymptomatic cases, the lack of mass testing, the lack of testing for suspicious deaths and the collapse of the health system, which inflates the mortality rate. From an epidemiological point of view, little is still known about SARS-CoV-2, and it is extremely difficult to compare this virus to human coronaviruses that cause mild and moderate disease or to those that are highly pathogenic and virulent, such as SARS-CoV and MERS-CoV [5, 6].

The coronavirus pandemic’s life-altering effects are likely to result in lasting physical and mental health consequences for many people, particularly those from vulnerable populations. In this sense, the extent and significance of the present epidemic in the Brazilian Amazon is in need of investigation.

Since the elimination of beta-coronavirus circulation requires a minimum herd immunity (indications 50–66%) [7], the information for which is still unknown at the local, national or global levels, conducting seroepidemiological and surveillance studies on SARS-CoV-2 in geographic areas such as the Amazon is extremely important, as it will allow for the assessment of the prevalence and titre of antibodies anti-SARS-CoV-2, mortality and case fatality rates and the epidemiological aspects of risk of exposure in communities from different population strata, such as *riberinhos* (riverain communities), *quilombola* (Afro-descendant communities) and indigenous peoples, providing an improvement in the decision-making of future epidemics.

It is important to note that recent study indicates that antibody response offered after infection with the SARC-CoV-2 may not be long enough [8]. If it can be influenced by type of serological assay, is still uncertain; but certainly it could impact the interpretation of seroepidemiological data, as well as, the level of exposure of the population to a second wave of infection. Thus, aiming to avoid bias, we propose that seroepidemiological studies should use methods with high sensibility and specificity, as enzyme-linked immunosorbent assay (ELISA) and immunometric quimioluminescence essay, in order to minimize any interference on antibody prevalence calculation. Furthermore, follow-up assessments of individuals in the population could tell us what is the average time of presence of antibodies in the blood and, thus, estimate the level of susceptibility to a new possible epidemic wave.

### The COVID-19 and the Amazonian peoples

The different Amazonian communities face distinct health and cultural challenges amid the pandemic. They have varied social and cultural dynamics mainly as urban and nonurban traditional populations are compared, which can interfere or not interfere with the dynamics of the dissemination of SARS-CoV-2. Thus, it is necessary to map both social and cultural behaviors to help understand the extent and meaning of the Coronavirus Disease 2019 (COVID-19) epidemic in the Brazilian Amazon. Furthermore, Amazonian peoples, especially those residing in remote areas, are continuously exposed to different pathogens endemic in the Amazon rainforest, and until now, we are not aware whether co-infection with those other pathogens could drive SARS-CoV-2 infection and clinical progression to COVID-19.

The number of confirmed cases and deaths by COVID-19 in municipalities located within the Special Indigenous Sanitary Districts (DSEIs) are higher than several areas other than the South and Southeast regions of the country. The circulation of indigenous people to regional centers where health access services, trade activities and some social interactions can be the reason for increasing the vulnerability of this population, a number that tends to increase due to the internalization of the epidemic to epidemiologically closed or semi-closed communities. According to the National Committee for Indigenous Life and Memory, a third of Brazilian indigenous populations, a total of 111 groups (44 ethnicities), would have already been affected by COVID-19, with more than 7000 infected and 300 deaths; this led the National Council of Justice (CNJ) of Brazil to create an observatory to monitor the protection of indigenous peoples [9].

Researchers from the Oswaldo Cruz Foundation (FIOCRUZ) and the Getulio Vargas Foundation (FGV) produced a report on the risk of COVID-19 spreading among indigenous populations, according to the geographic and sociodemographic vulnerability of this segment. The study suggests that COVID-19 represents great challenges for indigenous communities, health authorities and the whole of society, in order to promote actions to protect them against the virus, aiming to avoid major social health impacts [10].

Indigenous populations are thought to be more vulnerable to new infectious agents because of their genetic inability to respond immunologically in their initial contact with new infections. Additionally, these groups are made more vulnerable to the infection by SARS-CoV-2 within the presence of several co-infections and diseases present, including tuberculosis, malaria, human immunodeficiency virus (HIV-1), human T-lymphotropic virus (HTLV), *Chlamydia trachomatis, Treponema pallidum*, besides other social and environmental factors such as the lack of potable drinking water and malnutrition [11, 12]. Thus, moderate and severe cases of COVID-19 are expected to reach Indians communities due to scant living and health conditions.

Quite differently from urban populations, indigenous peoples historically are submitted to profound discrimination based on ethnicity, poverty and language; and preventive measures against the virus are not everyday practices in indigenous communities. Seroepidemiological surveys in these communities should assess a representative sample of the population (with adequate sample size), perform an adequate collection of sociodemographical data (sex, age, cultural habits and social behavior) and use serological tests with high sensitive and specific. It will increase the level of information about the present epidemic (prevalence, mortality and case fatality rates, and sociodemographic vulnerability), resulting in stronger ability to persuade the population to adhere to prevention and control measures against the virus, and establishing specific recommendations for a possible future reemergence of the virus in the Brazilian Amazon region.

## Conclusions

Seroepidemiological study associated with the sociocultural understanding of the different Amazonian indigenous communities will indicate the current level of individual susceptibility that may sustain a second wave of the epidemic and who should be the target for future vaccines. It is worth noting that comprehensive seroepidemiological information is expected to be provided, aiming the immediate use in decision-making and in the creation of government health policies. In addition to seroepidemiological surveillance studies, is paramount importance that the Brazilian government creates a detailed contingency plan that presents measures for the prevention and control of the epidemic in these communities, as well as supply the access to diagnosis, drinking water, hygiene material, emergency financial aid and Intensive Care Unit beds.

## Data Availability

Not applicable.

## Abbreviations

SARS-CoV-2: Severe acute respiratory syndrome coronavirus 2
WHO: World Health Organization
HCoVs: Human Coronavirus
SARS-CoV: Severe Acute Respiratory Syndrome Coronavirus
MERS-CoV: Middle East Respiratory Syndrome Coronavirus
COVID-19: Coronavirus Disease 2019
DSEIs: Special Indigenous Sanitary Districts
CNJ: National Council of Justice
FIOCRUZ: Oswaldo Cruz Foundation
FGV: Getulio Vargas Foundation
HIV: Human immunodeficiency virus
HTLV: Human T-lymphotropic virus
